# Effect of Microwave Irradiation on the Dielectric Characteristics of Semi-Conductive Nanoparticle-Based Nanofluids: Progress towards the Microwave Synthesis

**DOI:** 10.3390/mi14061194

**Published:** 2023-06-03

**Authors:** S. Raja, G. Koperundevi, Muthusankar Eswaran

**Affiliations:** 1 Department of Electrical and Electronics Engineering, National Institute of Technology Puducherry, Karaikal 609609, Puducherry, India; sankaranraja@rediffmail.com; 2 Department of Biology and Biological Engineering, Chalmers University of Technology, 412 96 Gothenburg, Sweden

**Keywords:** semi-conductive nanofluid, dielectric properties, titanium dioxide nanoparticle, zinc oxide nanoparticle, microwave synthesis

## Abstract

Studies on dispersing nanoparticles in base fluid to elevate its essential and critical properties have evolved significantly in the recent decade. Alongside the conventional dispersion techniques used for nanofluid synthesis, microwave energy at 2.4 GHz frequency is irradiated onto the nanofluids is experimented with in this study. The effect of microwave irradiation on the electrical and thermal properties of semi-conductive nanofluids (SNF) is investigated and presented in this article. Titanium dioxide and zinc oxide are the semi-conductive nanoparticles used for this study to synthesize the SNF, viz., titania nanofluid (TNF) and zinc nanofluid (ZNF). Flash and fire points are the thermal properties verified, and dielectric breakdown strength, dielectric constant ($\varepsilon_{r}$), and dielectric dissipation factor (tan δ) are the electrical properties verified in this study. AC breakdown voltage (BDV) of TNF and ZNF is improved by 16.78% and 11.25%, respectively, more than SNFs prepared without microwave irradiation. Results justify that the synergetic effect of stirring, sonication, and microwave irradiation in a rational sequence (microwave synthesis) exhibited better electrical and unaltered thermal properties. This microwave-applied nanofluid synthesis could be a simple and effective route to prepare the SNF with improved electrical properties.

## 1. Introduction

The concept of nanofluids has found a broad range of applications ever since its conception by Choi [1], while many experimental studies and investigations in various fields have justified its effectiveness in performance improvement of nanofluid systems [2,3,4,5,6]. Rafiq [7] has produced a thorough review of transformer oil-based nanofluids, which included several attributes such as types of nanoparticles, base fluids and nanofluids, types of synthesis and dispersion techniques, and its electrical, thermal, and physiochemical properties. Yu [8] has made an effective review of the recent progress, underlying mechanisms, characterization techniques, research challenges, and strategies in improving the dispersion stability of the water- and oil-based nanofluids used in thermal conductivity applications. The major challenge in nanofluid synthesis lies in dispersing the nano-sized solid particles in the base fluid to attain a homogeneous colloid, to ensure its long-term stability. A well-dispersed nanofluid is an essential prerequisite that decides its added functionalities, improvised properties, and enhanced performances. However, fluid stability is a fundamental, long-standing issue yet to be addressed to realize its potential for industrial-scale real-time applications.

To overcome the dispersion stability issue to a certain extent, several researchers used conventional dispersion techniques like mechanical stirring, ultrasonic treatment, and chemical methods of surface modification either separately or combined. Asif et al. [9] presented a detailed review that consolidated the research on various metal oxides and carbon nanotube (CNT) nanofluids and that experimented with the effect of sonication duration on its thermo-physical properties. This review found that an optimum sonication duration yields a better dispersion of nanoparticles with reduction in particle sizes, consequently leading to enhanced nanofluid stability and other thermo-physical properties. Several types of research were reported to achieve an effective dispersion of functionalized nanoparticles through ultrasonic probe sonication combined with stirring [10,11,12,13].

Transformer oil-based nanofluid synthesis is mainly focused on enhanced electrical and thermal properties with the conservative usage of dispersion techniques like stirring and ultrasonic homogenization. Yu-zhen Lv investigated the effect of dispersion techniques on the stability of functionalized TiO_2_ nanoparticles in transformer mineral oil and proposed the combination of stirring and bath sonication found to have an efficient dispersion with the maximum value of dielectric breakdown strength. Despite the higher sonication energy, the ultrasonic probe immersed in the fluid is reported to affect the adsorption balance of the surfactant oleic acid [14]. The synthesis procedure of titania-based transformer nanofluid through mechanical stirring and probe sonication was performed with the optimized levels of TiO_2_ and surfactant cetyl trimethyl ammonium bromide (CTAB) dispersed in transformer mineral oil. The quantity of titania nanoparticles and surfactant loading in mineral oil was optimized systematically by verifying its superior values of corona inception voltage and the AC and DC dielectric breakdown voltage [15]. Aside from the application type and user’s awareness of combining the techniques, no standards have yet been defined to realize the optimal combination to attain the superlative dielectric BDV values with an elated dispersion strength.

Research has revealed that the microwave route of nanoparticle synthesis ensures a better dispersion in comparison to the conventional methods such as sol-gel, chemical vapor deposition, hydrothermal, solvothermal, etc. Accelerated reaction rates and altered synthesis yields such as particle size reduction [16], particle distribution [17,18], and enhanced purity [19] and physiochemical properties [20] have been observed in microwave-assisted synthesis. Such improved parameters of microwave heating compared to the conventional heating processes at similar temperatures have led to speculations about the existence of non-thermal effects in microwave synthesis chemistry [21]. Apart from reducing the processing time from hours to minutes with rapid and uniform heating, this microwave irradiation has proven to be a simple and effective method to synthesize nanostructures, with the commonly used operating frequency being 2.45 GHz [21,22,23,24].

Such attractive features of the microwave-based method in nanoparticle synthesis caused the authors to investigate the same in nanofluid synthesis. Based on that, the microwave irradiation in the nanofluid synthesis process is experimentally investigated in this work in order to verify the enhancement of the dielectric and thermal behavior of transformer oil-based nanofluids. Hence, the objectives studied are: 1. To recognize an effective synthesis procedure with the application of microwaves to prepare the semi-conductive nanofluids (SNF), with the superlative combination of dispersion techniques in a rational sequence. 2. To investigate the electrical and thermal properties of SNF prepared through the microwave synthesis procedure. The electrical properties of an insulating liquid can be addressed by its dielectric behavior, hence the dielectric breakdown strength, dielectric permittivity, and dielectric dissipation factor of the TNFs are investigated in this work. Flash point and fire point are the physical property of an insulating liquid that determines its thermal volatility, hence it is verified for TNFs in this work.

## 2. Material, Methods, and Experimental Setup

For experimentation purposes, the commercially available TiO_2_ nanoparticles (Anatase, spherical, average particle size 10–20 nm) and zinc oxide nanoparticles (10–40 nm) were purchased from Nano Research Lab (NRL) India. Analytical grade cationic surfactant CTAB and non-ionic surfactant Span80 (sorbitan monooleate) were procured from Sisco Research Laboratories (SRL), India. Commercial purpose transformer mineral oil was procured from APAR Industries, India. The solvent cyclohexane (for HPLC, 99.9%) used for mineral oil and other SNF samples, was procured from SRL, India, for the ultraviolet-visible (UV-Vis) spectroscopy studies.

**Figure 1:**
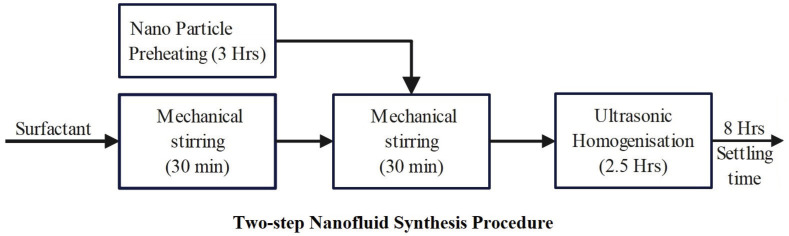
Two-step nanofluid synthesis procedure.

**Figure 2:**
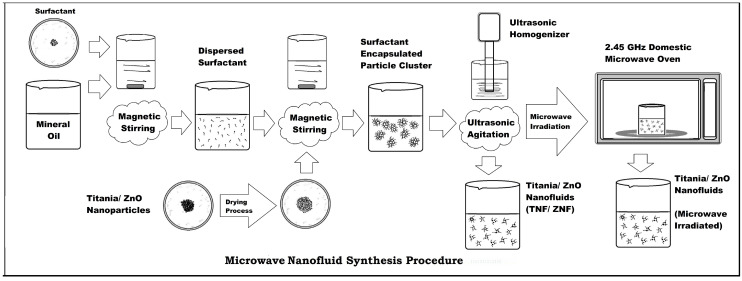
Process flow-diagram of microwave nanofluid synthesis.

The nanofluid samples used in this study for experimentation purposes were prepared by the two-step method of nanofluid synthesis, as shown in Figure 1. Initially, the semi-conductive nanoparticles (TiO_2_/ZnO) were dried in a heating chamber for about 3 h to eliminate moisture. Before it was dispersed in mineral oil, the surfactant CTAB/Span80 was mixed with mineral oil by mechanically stirring it in a magnetic stirrer for 30 min. Later, the nanoparticles were added to it through mechanical stirring for 30 min and subsequently dispersed using the ultrasonic homogenizer probe (750 W, 20 KHz, Sonics Vibra-cell) for about 2.5 h. It was then irradiated with microwave energy at 2.45 GHz frequency in a commercial microwave oven to progress to the microwave synthesis. The process flow of the microwave nanofluid synthesis procedure is shown in Figure 2 as a pictorial representation.

The UV-Vis absorption spectrum was verified for evaluating the dispersion strength of the prepared SNF samples. Spectroscopy studies were carried out using a UV-Vis double-beam spectrophotometer (Analytical Technologies). Cyclohexane was used as the solvent for the oil samples and as a reference solution in the quartz cuvettes of the spectrophotometer, respectively.

The dielectric breakdown strength, dielectric constant, and dielectric dissipation factor of the SNFs were investigated in this work to evaluate the electrical properties of SNF samples, whereas the flash point and fire point were measured to assess the thermal volatility of SNF samples.

The AC breakdown voltage test was performed using the Motorized Oil Breakdown Voltage Test System (LOBDV-100, Power Electronical, India), which has an AC high-voltage (maximum up to 100 KV) generating unit and an oil test cell as per IEC 60156 standards. Hemisphere electrodes with 2.5 mm spacing were used in the test cell. The dielectric constant and tan δ of the oil samples were measured using the automatic dielectric constant, tan delta, and resistivity test set (ADTR-2K PLUS, Eltel Industries, India). The oil sample was filled in a three-terminal test cell (IEC-60247 standards), and the test was performed under a pre-programmed test sequence. Flash point and fire point of the oil samples were measured in a Pensky–Martens flash point apparatus as per ASTM D971 standards, with a temperature increase rate of 5 to 6 degrees Celsius per minute.

## 3. SNF Synthesis

The SNF samples were prepared by dispersing the optimized quantity of titania/zinc oxide nanoparticles and surfactant CTAB/Span80 in mineral oil used for insulation and cooling the transformers in high voltage applications. As previously studied [15], 0.05 g/L of titania nanoparticle and 0.5 mg/L of CTAB were identified as optimal quantities based on the higher dielectric BDV obtained, and the same quantity was used in this work for TNF synthesis.

**Figure 3:**
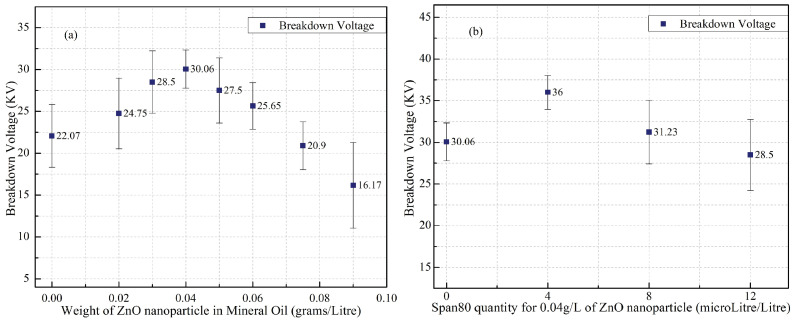
(**a**) BDV of ZO for different concentrations of ZnO nanoparticles, (**b**) BDV of SZO for different quantities of surfactant Span80.

To synthesize ZNF in this work, the quantity of ZnO nanoparticles and Span80 had to be optimized systematically at first. Therefore, a series of samples were prepared with different concentrations of ZnO nanoparticles ranging from 0.02 g/L to 0.09 g/L. A two-step synthesis procedure as shown in Figure 1 was used to prepare the samples, and their dielectric BDV values were tested and shown in Table 1 and Figure 3a.

The sample with the highest BDV value was chosen as the optimum quantity of ZnO nanoparticle to be dispersed in mineral oil. From the 7 samples prepared with different ZnO weights of 0.02, 0.03, 0.04, 0.05, 0.06, 0.075, and 0.09 g/L, the 0.04g/L ZnO exhibited the highest BDV magnitude enhancement of 36.2%. With that quantity being fixed as optimum, the next series of samples (ZNFs) were prepared with different Span80 concentrations of 4, 8, and 12 µL added. The dielectric BDV values were verified for the same and are shown in Table 2 and Figure 3b.

The AC breakdown voltage test was conducted as per IEC 60156 standards with 10 breakdowns for each sample. Discarding the two lowest and the two highest values, the remaining six values were used to calculate the mean AC breakdown voltage of the samples. The sample that exhibited the highest BDV value was considered as the optimum concentration of ZnO nanoparticles and Span80 for the ZNF synthesis in this work. Hence, 0.04 g/L of ZnO nanoparticles and 4 µL of surfactant Span80 were fixed as the optimum quantities for this part of the study.

The different types of TNF and ZNF samples prepared for this study and the naming conventions used in this manuscript are listed in Table 3.

The sample type TO was prepared by dispersing 0.00562 wt.% (0.05 g/L) of TiO_2_ nanoparticles in mineral oil through magnetic stirring for 30 min and then sonication for 2.5 h with the help of an ultrasonic probe homogenizer. In the case of CTO, the surfactant CTAB with 1% weight titania (0.5 mg/L) was mixed with mineral oil through stirring for 30 min, before adding TiO_2_ nanoparticles into it. The obtained nanofluid was irradiated with microwave energy at 2.45 GHz frequency in a commercial microwave oven to prepare the CTMW sample. A similar pattern was followed for ZNF synthesis, with 0.04 g/L of ZnO nanoparticles and 4 µL of surfactant Span80 dispersed in mineral oil.

Research into microwave-assisted synthesis has revealed that microwave irradiation duration has a certain impact on nanoparticle synthesis yield, such as particle size and its dispersivity while preparing nanofluids [25]. Hence, the authors intended to identify the optimal irradiation time through verifying the dispersivity of TNF for different microwave irradiation times. A series of CTMW samples were prepared with different irradiation times of 60, 90, 120, 180, and 240 s, keeping in mind that the temperature of the sample did not exceed the transformer’s top oil temperature of 65 °C. For all CTMW samples prepared at different time durations, the microwaves were applied intermittently onto the sample, with 60 s ON time and 20 s OFF time to avoid rapid heating of the sample.

## 4. Dispersivity Evaluation

As mentioned earlier, dispersion studies were conducted with the help of a UV-Vis double beam spectrophotometer. Dispersion strength of the nanofluid was evaluated by analyzing the UV-visible absorption spectrum of TNF samples prepared in this work. UV-visible spectral analysis is a simple, reliable, and widely used technique to analyze nanofluid dispersion. The optical absorption intensity of the sample when diluted appropriately with a suitable solvent is linear to the nanoparticle concentration in the fluid, and the absorption peak decreases correspondingly as the fluid becomes destabilized.

**Figure 4:**
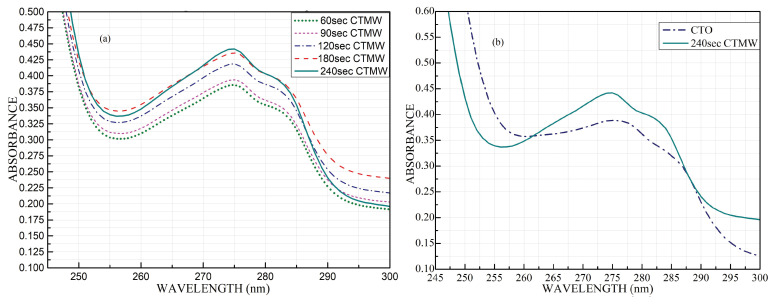
(**a**) UV-visible spectra of CTMW samples for different irradiation times, (**b**) UV-visible spectra comparison of CTO and CTMW samples.

The UV-visible spectra of the effect of microwave irradiance duration on CTMW samples are shown in Figure 4a. It can be seen that all the CTMW samples exhibit their characteristic spectra with a typical absorption peak at the wavelength of 275 nm. It can also be observed that the higher the irradiation time, the higher the intensity of the absorbance peak, which could be a measure of observing the dispersion process. A substantial increase in the UV absorbance observed as the irradiance time changes from 60 s to 240 s demonstrates a better dispersion of nanoparticles in the colloid [6,26,27,28]. Therefore, it is inferred from the results that the longer duration of microwave irradiation leads to an enhanced homogenized suspension, and hence better homogeneity is achieved with 240 s of microwave irradiance.

A comparison of absorption spectra of the 240 s CTMW sample and the CTO sample is shown in Figure 4b. The significant difference in the absorbance peaks implies that the particles are well-dispersed in TNF irradiated with microwaves. This ascertains the difference in the dispersion strength of the CTO and CTMW samples and establishes the conclusiveness of the microwave effect on the nanofluid dispersion process. Therefore, the UV-Vis absorption spectra of these two samples provide a better realization of the impact of microwaves in the dispersion process and their potential as an effective dispersion mechanism for nanofluid synthesis.

**Figure 5:**
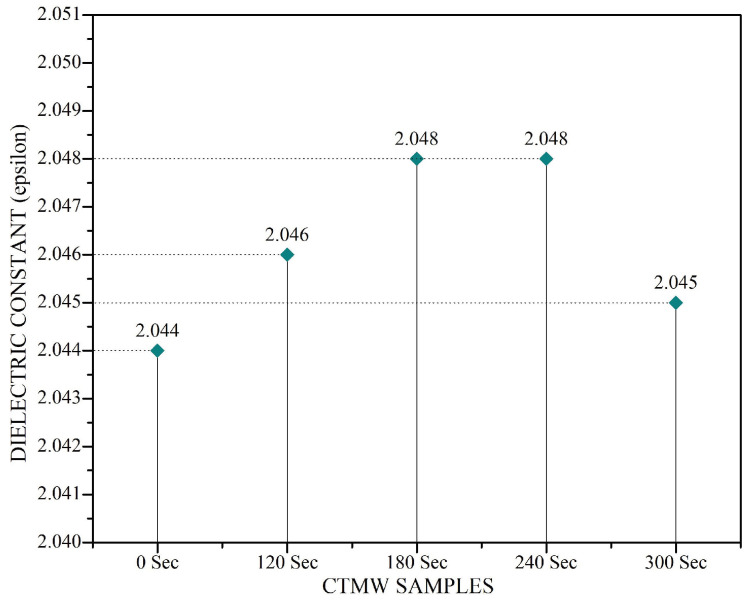
Permittivity of CTMW samples for different microwave irradiation times.

Along with the dispersivity evaluation results discussed above, it was further decided to verify the microwave effect on the dielectric permittivity of the CTMW sample. A series of CTMW samples were prepared by irradiating microwaves for different time durations of 120, 180, 240, and 300 s, and the dielectric permittivity was measured. A certain modification in dielectric constant values was observed in the CTMW samples as the duration of microwave irradiation increased. The observations were plotted, and the graph plot is shown in Figure 5.

## 5. Results and Discussion

### Dielectric and Thermal Studies

**Figure 6:**
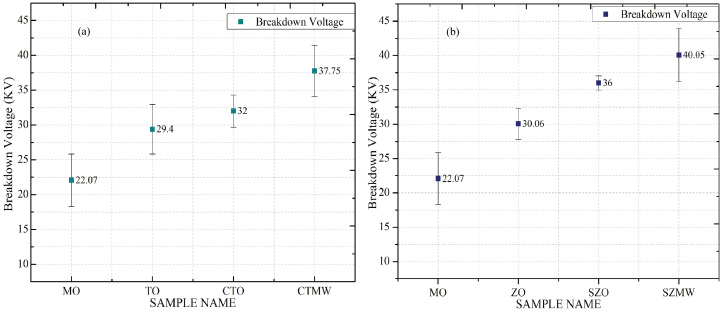
(**a**) Dielectric breakdown voltage of different titania nanofluid samples, (**b**) Dielectric breakdown voltage of different zinc nanofluid samples.

**Figure 7:**
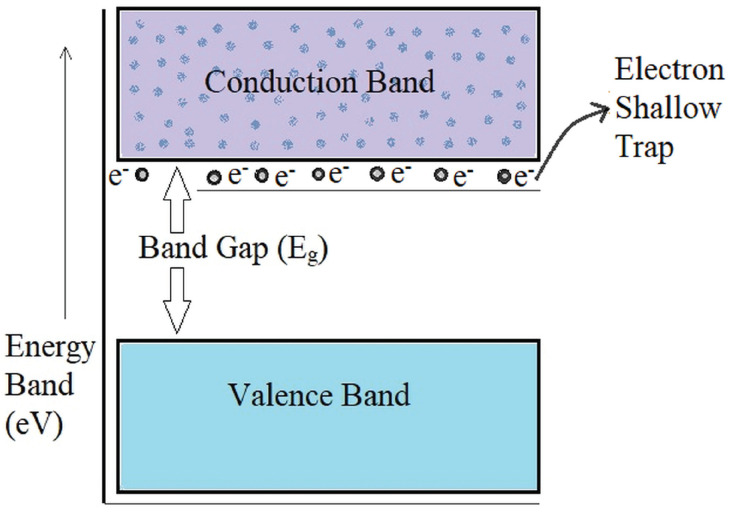
Schematic of the electron shallow trap sites.

**Figure 8:**
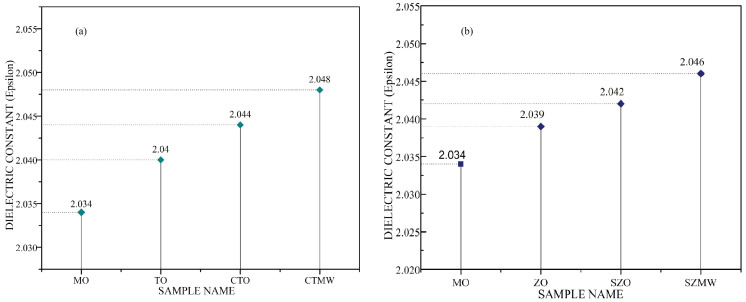
(**a**) Dielectric permittivity of different titania nanofluid samples, (**b**) Dielectric permittivity of different zinc nanofluid samples.

**Figure 9:**
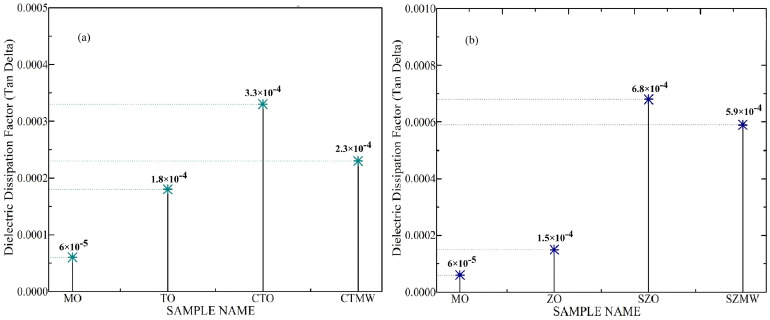
(**a**) tan δ value of different titania nanofluid samples, (**b**) tan δ value of different zinc nanofluid samples.

The experimentally generated values of breakdown voltage, dielectric constant, (tan δ), flash point, and fire point obtained for the SNF samples are presented together in Table 4, and their respective graphs are plotted separately and shown in Figure 6, Figure 7, Figure 8 and Figure 9. Figure 6 shows the graph plotted for the AC BDV magnitudes of the titania and zinc nanofluids. The experimental results show that both SNFs exhibit the maximum value of dielectric BDV when irradiated with microwaves. It also shows that the BDV magnitudes obtained for the TNF and ZNF with microwave irradiation are 16.78% and 11.25%, respectively, higher than that of the SNFs prepared without microwave irradiation.

The BDV magnitude is enhanced with the addition of TiO_2_/ZnO nanoparticles in MO and further improved with the addition of the surfactant CTAB/Span80 into it. The higher BDV value of the SNFs is attributed to the suppression of corona discharge inception and its propagation in the fluid. Yuefan Du et al. proposed that the semi-conductive nanoparticles dispersed in the mineral oil trap the fast-propagating electrons from the streamer, which triggers the insulation breakdown to form the current conduction path between electrodes. These electrons are trapped at the molecular level of the nanostructures and then get converted into slow-moving negative charges by losing their kinetic energy. This molecular level of electron trapping in the semi-conductive nanoparticles happens in the energy states that are created in the bandgap edges, called shallow traps. A schematic of the electrons trapped in the shallow trap sites is shown Figure 7. This type of charge transport process is a predominant mechanism observed with the semi-conductive nanoparticles that aids in BDV magnitude enhancement [29,30,31,32]. Further improvement of BDV is observed in the results with the addition of surfactant CTAB and Span80 along with the TiO_2_ and ZnO nanoparticles, respectively. According to Mansour et al., the surfactant molecules with the hydrophilic head and hydrophobic tail encapsulate the nanoparticles dispersed in mineral oil. With the molecule head being attracted to the particle surface, its tail is projected normally in an oil medium, forming an intact zone at the oil–particle interface. The electrons lose their energy through this zone, which results in an increased BDV magnitude [33].

The dielectric permittivity values of the different TNF and ZNF samples are shown in Figure 8. The results show that the permittivity values of the TO and ZO samples are higher than those of virgin mineral oil and that the main contribution is the permittivity of the nanoparticles dispersed in it. Electric permittivity holds the information on the polarization of a dielectric material that is correlated with the number of electric dipoles and the molecular polarizability of the medium [34]. A nanoparticle dispersed in dielectric liquid will acquire a charge through different mechanisms and form an EDL around the particle surface. Jin et al. proposed that the nanofluid attains three kinds of polarization in its medium when supplied with an external electric field that increases the overall polarization of the bulk fluid. Polarization of oil molecules, the nanoparticle’s inner polarization, and the orientation of the charged nanoparticles are the major polarization mechanisms that occur. Therefore, the net charge created in the particle fluid interface increases the polarisation of bulk fluid, and hence the dielectric permittivity [35,36]. According to Sima et al., this interfacial polarization modifies the electrodynamics of the SNF medium and leads to the formation of the potential well around the particle surface. It traps the fast-propagating ions and electrons and converts them to slow charges, leading to the enhanced BDV of SNFs [37,38].

Microwaves irradiated on the dielectric medium do have a microscopic level of interaction with their constituent atoms, and free electrons induce polarization mainly due to the orientation of their dipoles and free electrons [27]. This results in a dielectric constant improvement that could further impact the enhancement in BDV magnitude, as discussed earlier. This is evident from the experimental results, which verified the enhanced dielectric permittivity and BDV for the microwave-applied samples (CTMW and SZMW). With the addition of surfactant, the slight increment observed in the dielectric constant of the CTO sample is caused by polarization due to surfactant encapsulation. Farade et al. [39] proposed the relative permittivity model of the nanofluid system, with the surfactant contributing the orientation polarization of the nanoparticle and hence the dielectric permittivity.

Figure 9 shows the tan delta values of the various TNF and ZNF samples. It can be noted that the dielectric dissipation factor of TO and ZO samples is higher than that of MO and is subsequently increased by adding surfactant CTAB and Span80 respectively. Ming dong et al. indicated that the addition of nanoparticles and the presence of EDL contribute slightly to the conductivity of mineral oil and further increase with the increase in nanoparticle concentration. Under the applied electric field, the charged nanoparticle and its surrounding ion cloud of EDL undergoes the electrophoresis effect and forms electrophoresis conductivity, which depends on the base fluid’s viscosity [35]. Such an enhanced conductivity with the addition of nanoparticles would slightly increase the dielectric loss of nanofluid medium.

**Figure 10:**
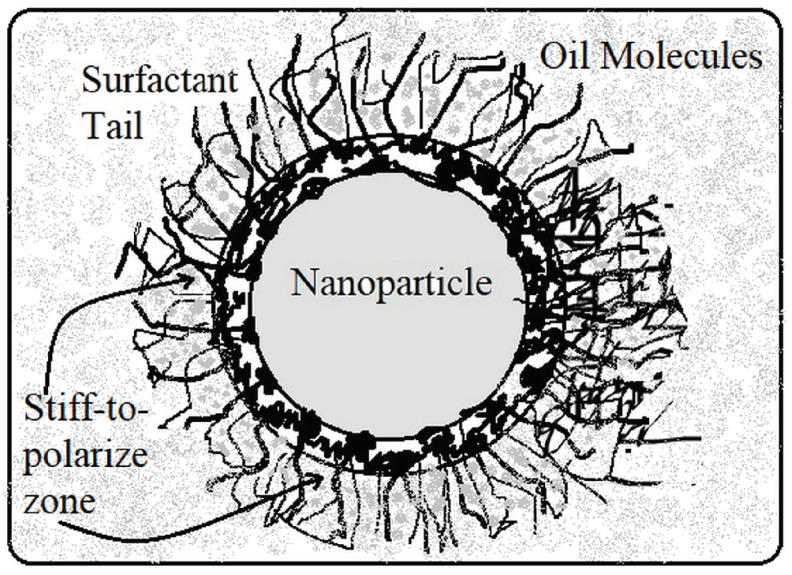
Stiff to polarize zone of the surfactant encapsulated nanoparticle.

According to Mahidhar et al., the increment in the tan delta value for the surfactant addition in SNFs is attributed to the interfacial layer that it normally forms on the oil molecules. Figure 10 shows the surfactant encapsulated nanoparticle dispersed in oil molecules with tails projected normally, forming the ‘stiff to polarize’ zone. The oil molecules present in this zone are rigid enough to become polarized by the externally applied voltage. Therefore, the torque created from the electric field required to orient the dipole moments of oil molecules present in this ‘stiff to polarize’ layer is a bit larger. Thus, higher potential energy is required to orient these dipole moments of the interfacial layer. More energy lost in the medium causes the increment in the tan delta values [40,41]. However, in the case of microwave-irradiated SNFs, the obtained experimental results have slightly decremented tan delta values compared to CTO and SZO, but they are still higher than TO and ZO.

**Figure 11:**
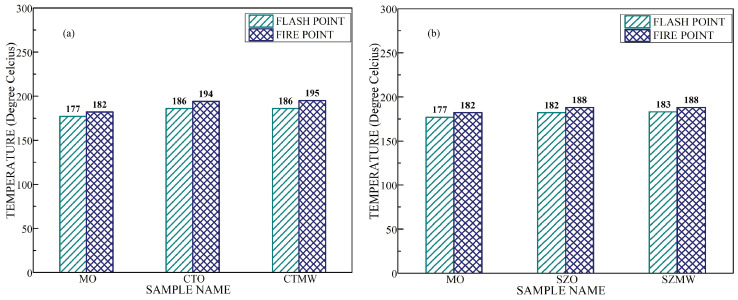
(**a**) Flash point and fire point temperatures of different titania nanofluid samples, (**b**) Flash point and fire point temperatures of different zinc nanofluid samples.

Figure 11 shows the flash point and fire point temperature values of the oil samples. It shows higher values for the TNF and ZNF when compared with virgin mineral oil and remains unchanged for the microwave-irradiated sample. Enhancement in thermal characteristics for the addition of TiO_2_/ZnO nanoparticles is attributed to the heat transport mechanism through phonons, which is created within the nanostructures due to the Brownian motion of oil molecules. The flash point and the fire point remain unchanged for the CTO and CTMW, as the Brownian motion of oil molecules tends to disrupt the polarization effect induced by microwaves [32,42].

## 6. Conclusions

The microwave route of the nanofluid synthesis procedure has been experimented with in this work for semi-conductive nanoparticles like titania and zinc oxide. The major conclusions derived from this study are as follows:

- The dispersing quantity of ZnO nanoparticles and surfactant Span80 for ZNF preparation are optimized systematically based on BDV enhancement. MO dispersed with 0.04 g/L of ZnO nanoparticles and 4 µL/L of surfactant Span80 provided 63.12% of BDV enhancement compared to mineral oil and is considered an optimal concentration from this study.
- A dispersion study was conducted for the CTMW sample to verify and fix the microwave irradiation time on the nanofluids. From the higher absorption peak of the UV-Visible absorption spectra of five CTMW samples with different irradiation times, the 240-s sample was inferred as providing a better dispersion; therefore, this measurement was fixed as the optimal irradiation time.
- Dielectric and thermal properties were investigated for the SNFs with and without applying microwaves. TNF and ZNF irradiated with microwaves for 240 s improved their BDV by 16.78% and 11.25%, respectively, which is attributed to their improved dielectric permittivity. Permittivity enhancement is explained in terms of polarization phenomena induced by the microwaves interacting with the dielectric medium.
- From the conducted experiments, the flash point and fire point temperature values of the TNF and ZNF did not show any modification with the irradiation of microwaves.
- Increased tan delta values of the SNF with the surfactant addition were slightly reduced with the application of microwaves. However, the modification in tan delta values requires a clearer study owing to the inconsistency in the reported results from various research studies.

The proposed microwave synthesis procedure is verified for its good dispersivity and incremented dielectric behavior for the semi-conductive nanoparticle-based nanofluids. Therefore, this microwave route could effectively contribute to framing a standard two-step procedure for nanofluid synthesis.

## Figures and Tables

**Table 1:** AC BDV value of sample series with different ZnO concentrations.

S. No.	Quantity of ZnO Nanoparticles Dispersed in Mineral Oil (g/L)	Mean AC BDV with Standard Deviation (KV)	BDV Enhancement (in %)
1	0	22.07 ± 3.75	0
2	0.02	24.75 ± 4.23	12.14
3	0.03	28.5 ± 3.72	29.13
4	0.04	30.06 ± 2.28	36.20
5	0.05	27.5 ± 3.89	24.60
6	0.06	25.65 ± 2.78	16.22
7	0.075	20.9 ± 2.86	−5.3
8	0.09	16.17 ± 5.1	−26.73

**Table 2:** AC BDV value of sample series with different Span80 concentrations.

S. No.	Quantity of Surfactant Span80 with 0.04 g/L ZnO Nanoparticles Dispersed in Mineral Oil (µL/L)	Mean AC BDV with Standard Deviation (KV)	BDV Enhancement (in %)
1	0 µL/L	30.06 ± 2.28	0
2	4 µL/L	36 ± 2.03	19.76
3	8 µL/L	31.23 ± 3.83	3.89
4	12 µL/L	28.5 ± 4.26	−6.99

**Table 3:** Naming conventions for the TNF and ZNF samples used.

Name of Sample	Sample Type
MO	Virgin mineral oil
TO	Virgin mineral oil + TiO_2_ nanoparticles
CTO	Virgin mineral oil + surfactant CTAB + TiO_2_ nanoparticles
CTMW	Microwave-irradiated CTO
ZO	Virgin mineral oil + ZnO nanoparticles
SZO	Virgin mineral oil + Span80 + ZnO nanoparticles
SZMW	Microwave-irradiated SZO

**Table 4:** Dielectric and thermal properties of the SNF samples used.

Name of Sample	Dielectric Properties				Thermal Property	
	Breakdown Voltage (kV)		Relative Permittivity	Tan Delta	Flash Point (°C)	Fire Point (°C)
	Mean	SD				
MO	22.07	3.75	2.034	0.00006	177	182
TO	29.4	3.57	2.04	0.00018	–	–
CTO	32	2.3	2.044	0.00033	186	194
CTMW	37.75	3.65	2.048	0.00023	186	195
ZO	30.06	2.27	2.039	0.00015	–	–
SZO	36	1.03	2.042	0.00068	182	188
SZMW	40.05	3.85	2.046	0.00059	182	188

## Data Availability

Data is contained within the article.
